# Total Ankle Replacement Infections: A Systematic Review of the Literature

**DOI:** 10.3390/jcm12247711

**Published:** 2023-12-15

**Authors:** Renato Zunarelli, Michele Fiore, Gianluca Lonardo, Andrea Pace, Valentina Persiani, Massimiliano De Paolis, Andrea Sambri

**Affiliations:** Orthopedic and Traumatology Unit, IRCCS Azienda Ospedaliero-Universitaria di Bologna, 40138 Bologna, Italy; renato.zunarelli@ior.it (R.Z.); michelefiore.md@gmail.com (M.F.); gianluca.lonardo@gmail.com (G.L.); andrea.pace@ior.it (A.P.); valentina.persiani@aosp.bo.it (V.P.); massimiliano.depaolis@aosp.bo.it (M.D.P.)

**Keywords:** total ankle replacement, prosthetic joint infection, revision, two-stage, DAIR

## Abstract

Periprosthetic infection (PJI) after TAR is a serious complication, often requiring further surgery, including revision arthroplasty, conversion to ankle arthrodesis, or even amputation. This systematic review aims to summarize the current evidence on the management of TAR PJI and provide a comprehensive overview of this topic, especially from an epidemiologic point of view. Three different databases (PubMed, Scopus, and Web of Science) were searched for relevant articles, and further references were obtained by cross-referencing. Seventy-one studies met the inclusion criteria, reporting on cases of TAR PJI. A total of 298 PJIs were retrieved. The mean incidence of PJI was 3.8% (range 0.2–26.1%). Furthermore, 53 (17.8%) were acute PJIs, whereas most of them (156, 52.3%) were late PJIs. Most of the studies were heterogeneous regarding the treatment protocols used, with a two-stage approach performed in most of the cases (107, 35.9%). While the prevalence of ankle PJI remains low, it is potentially one of the most devastating complications of TAR. This review highlights the lack of strong literature regarding TAR infections, thus highlighting a need for multicentric studies with homogeneous data regarding the treatment of ankle PJI to better understand outcomes.

## 1. Introduction

Ankle osteoarthritis (OA) affects approximately 1% of the population [1]. Ankle arthrodesis (AA) has been considered the gold standard for the management of pain and deformity secondary to ankle OA for many years. However, this procedure has been linked with the development of OA in adjacent joints, resulting in further pain and disability, and with an approximately 10% non-union rate [2]. Total ankle replacement (TAR) has been increasingly used in clinical practice as an alternative to AA, in particular when other foot joints are involved by arthritis [3]. The preserved mobility of the ankle following TAR might be accompanied by a more successful functional outcome and the protection of adjacent joints from arthritis [4]. 

Nevertheless, no conclusive evidence exists as to which intervention provides the best outcomes for patients. In particular, some authors have advocated for higher complication rates for TAR compared to AA [5]. 

Periprosthetic infection (PJI) after TAR is a serious complication, often requiring further surgery, including revision arthroplasty, conversion to ankle arthrodesis, or even amputation.

The incidence of PJI after TAR ranges from less than 1% to more than 20% [6,7,8,9,10,11,12,13,14]. Unlike the hip joint, the ankle has a frail soft-tissue envelope, making infection following surgery an even more difficult problem to manage. The goals in treating the infection are first to eradicate it, and then to restore a painless functional limb [15]. Surgical options for the treatment of TAR PJI include debridement and implant retention (DAIR) in patients with acute PJI, one- or two-stage revision, or amputation [10,16]. Reconstruction after TAR revision (either single or two-stage) may be performed with a new TAR or AA.

However, unlike for patients with hip or knee arthroplasties, data on periprosthetic ankle infections are limited. In fact, due to the relative rarity of this condition, the few published articles are often small retrospective case series, with missing or highly heterogeneous data regarding PJI risk factors, causative microorganisms, infection type (early/delayed or late onset), treatments performed, and outcomes.

This systematic review aims to summarize the current evidence on the management of TAR PJIs and provide a comprehensive overview of this topic, especially from an epidemiologic point of view. 

## 2. Methods

This systematic review was conducted in accordance with the 2020 PRISMA guidelines (Preferred Reporting Items of Systematic Reviews) [17]. All studies (randomized controlled trials (RCT), prospective (PCCS) and retrospective comparative studies (RCCS), and prospective (PCS) and retrospective case series (RCS)) reporting on PJI of TAR were included. Biomechanical studies, cadaveric studies, “in vitro” studies, and animal model studies were excluded. Only articles in English published in a peer-reviewed journal were included. 

The criteria used to select articles allowed for us to extrapolate data about the outcomes of TAR, in particular the incidence of PJI and its treatment. Studies eligible for this systematic review were identified through an electronic systematic search of PubMed, Scopus, and Web of Science up to 31st August 2023. The search string used was as follows: (total ankle replacement OR total ankle arthroplasty) AND (periprosthetic joint infection OR outcomes OR failure). Articles without an abstract were excluded from the study. The articles were screened considering the relevance of titles and abstracts and by looking at the full-text of articles when the abstract provided insufficient information about inclusion and exclusion criteria. 

Articles that were considered relevant via electronic search were retrieved in full text, and a cross-referencing search of their bibliographies was performed to find further related articles. Reviews and meta-analyses were also analyzed in order to broaden the search to studies that might have been missed through the electronic search. All duplicates were removed, and all the articles retrieved were analyzed. After the first screening, records without eligibility criteria were excluded. 

Remnant studies were categorized by type, according to the Oxford Centre for Evidence-Based Medicine (OCEBM).

Each study was assessed by three reviewers independently; disagreement was resolved by the senior author. All the included studies were analyzed, and data related to topics of interest were extracted and summarized. 

In detail, the data extracted included the number of patients, number of PJI, mean age, type and timing of infections, data on PJI treatment, mean follow-up, and outcomes.

Cumulative PJI incidence was calculated considering only those studies that reported the whole TAR series. Series reporting only on a sub-cohort of failed TAR were excluded from this analysis. Recurrences and reinfections were defined by whether they were caused by the same or a different microorganism as in the first PJI, respectively.

The study is descriptive, and data are presented as total frequencies and percentages. The heterogeneity of most of the included studies did not allow any statistical analysis.

## 3. Results

**Figure 1:**
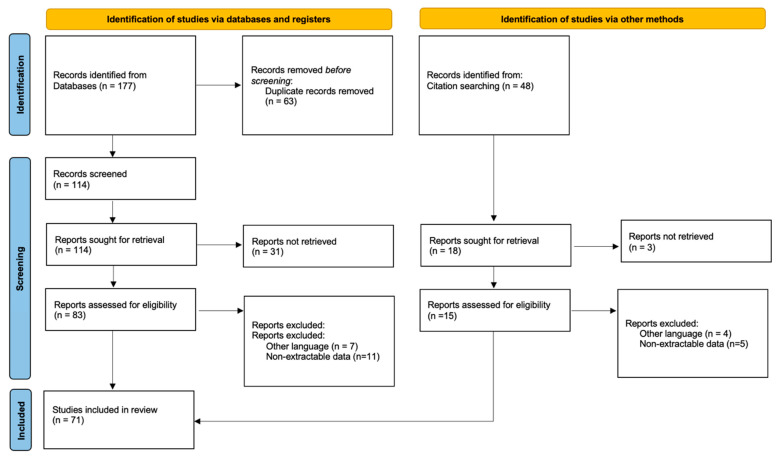
PRISMA flow diagram and the selection of studies.

A total of 177 studies were found through the electronic search, and 48 studies were added after cross-referenced research on the bibliographies of the examined full-text articles (Figure 1).

After a preliminary analysis, a total of 71 series reporting on PJI of TAR cases and their treatment were included in this systematic review (6 PCCS, 6 RCCS, 1 case report, and 58 retrospective case series).

A total of 10,662 TARs were retrieved (Table 1).

The mean age across all studies was 60.4 ± 10.5 years. The mean follow-up period was 48.4 months, ranging between 4 and 150 months. 

Overall, PJI was observed in 298 cases. Of these, 6 series [26,29,30,31,54,79] described sub-cohorts that included only PJI cases or prosthetic loosening. Thus, considering only the series including the whole TAR cohort, the mean incidence of PJI was 3.8% (range 0.2–26.1%). 

Among the included studies, PJI occurred at an average time of 31.3 months. In detail, 53 (17.8%) were acute PJIs, whereas most of them (156, 52.3%) were late PJIs. In addition, 22 (7.4%) infections were defined as acute hematogenous [18,25,50,52,84]. However, in 67 (22.5%) cases, PJIs were not classified according to the time of occurrence. 

Only 6 studies reported more than 10 PJIs. Moreover, most of them were heterogeneous regarding the treatment protocols used.

Nonetheless, cumulative data show that in most of the cases (107, 35.9%) a two-stage approach was performed. Its success rate has been reported to be very heterogeneous, with a generally lower success rate than in knee and hip PJI revision (Table 2).

Myerson et al. [18] reported on 7 PJIs treated with a staged revision out of a cohort of 19 infected TARs, with 3 recurrences of the infection. On the other hand, Kessler and Richter [11] reported a 100% rate of infection-free survival in 9 and 11 patients, respectively, treated with a two-stage revision procedure. 

In contrast, a one-stage procedure was used in only 68 cases (22.8%). In most of these, reconstruction was performed with an AA. 

DAIR was reported in 77 cases (25.8%). Even though a few reports did not report any failure after DAIR [28,37,38,41,49,53], the success rate of DAIR is generally scarce. Myerson [18] reported the results of 4 cases treated with DAIR, with a 100% failure rate, eventually requiring a two-stage revision. Similarly, Spirt et al. [85] reported an 80% rate of failure after DAIR in 5 cases of infected TARs. Lachman et al. [50] reported a 46% success rate when expeditious DAIR and polyethylene exchange with culture-appropriate antibiotics was performed. 

Five (1.7%) patients were treated with chronic antibiotic therapy without any surgery, whereas in sixty-seven cases the type of treatment was not reported. 

In 15 cases (5%) cement spacers ended up being the definitive treatment [30,86], whereas 22 cases (7.3%) required amputation for infection persistence. 

Most of the series did not provide accurate details about isolated pathogens. Nonetheless, among the available data, in most of the cases the microorganism isolated was *S. Aureus* (SA) (18 methicillin-sensible SA, 5 methicillin-resistant SA, and 8 did not specify whether SA was methicillin resistant). Other reported pathogens were coagulase negative: *Staphylococci* (18 cases), *E. coli* (2 cases), *S. viridans* (3 cases), *P. aeruginosa* (3 cases), *K. pneumoniae* (2 cases), *E. faecalis* (2 cases), *E. cloacae* (2 cases), *C. albicans* (1 case), *P. acnes* (1 case), *Peptostreptococcus* (1 case), *Citrobacter koseri* (1 case), *S. milleri* (1 case), *Diphtheroid* (1 case), *S. mitis* (1 case), *S. dysagalactiae* (1 case), *A. baumanii* (1 case), and *Corynebacterium* spp. (1 case).

In the majority of the cases (151, 50.7%) a novel TAR was after performed after prosthesis revision, while 72 (24.2%) patients had an AA. In 38 cases the definitive treatment was not reported. 

No information reporting the functional outcomes after the revision of a TAR for PJI were available. However, van der Heide et al. [13] identified the infection as an independent predictor of low clinical and functional outcomes in TAR.

## 4. Discussion

Total ankle replacement is increasingly used in ankle OA with progressively wider indications, and this leads to a consequent increase in complications [8]. 

The prevalence of PJIs after TAR occurred in just over 3.8% of implants included, which is consistent with that reported in prior studies [87,88]. Among the published series with 50 or more prosthesis, reported rates of infection following TAR range from 2% to 8.5% [9,12,27,89].

Acute PJI after TAR tends to be less common than chronic [12,18,19]. In the present review, only 53 PJIs were found to occur within the acute period, compared to 156 deemed as chronic PJIs. 

A wide range of possible treatments has been reported in the literature, which includes DAIR with or without polyethylene exchange, one- or two-stage revision arthroplasty, or even primary amputation.

Irrigation and debridement are usually performed in early postoperative PJI, more commonly associated with polyethylene exchange. However, outcomes from treating an infection with DAIR, polyethylene exchange, and retention of metal components in the total ankle revision literature are relatively scarce [90,91]. The lower efficacy of DAIR compared to other sites might be partially explained by the difficulties in accessing areas such as the posterior aspects of the gutters, which is unique to the anatomy of the ankle. Myerson et al. [18] reported the results of 4 cases treated with DAIR, with a 100% reinfection rate requiring a two-stage revision. Similarly, Spirt et al. [85] reported an 80% rate of failure after DAIR in 5 cases of infected TARs. Doets et al. [14] described one failure in a cohort of 5 infected TARs treated with open lavage combined with culture-specific systemic intravenous antibiotics. 

Recently, D’Errico et al. [92] observed that the eradication rates of DAIR with polyethylene exchange were slightly higher compared to DAIR alone because these steps theoretically lead to the most accurate debridement. Brage et al. reviewed 4 cases of irrigation and debridement, 2 of which also had polyethylene exchange in the face of acute hematogenous PJIs [19]. Both patients who underwent DAIR and polyethylene exchange retained their implants. Lachman et al. [50] reported a 46% success rate when expeditious DAIR and polyethylene exchange with culture-appropriate antibiotics was performed. However, they observed that patients who were symptomatic for longer and the presence of antibiotic-resistant bacteria both decrease the success of this operation. 

Most of the revisions of the implants were performed with a staged approach. A variety of different outcomes have been reported in the literature following two-stage revision. The eradication rate for two-stage exchanges in TAR infections was slightly lower than those usually reported for hip and knee PJIs. The different surgical site, with reduced a blood supply and more frequent wound healing problems, should be taken into account for this difference [67]. Myerson et al. [18] performed a staged revision arthroplasty in 7 patients out of a cohort of 19 infected TARs, with 3 recurrences of the infection. On the other hand, Kessler et al. [11] reported no failures among 9 patients treated with a two-stage revision procedure, while in the same series 21 PJIs were treated with retention of one or both components, resulting in an infection recurrence of 33%. Retention of one or both components might be considered in cases of an acute infection, only slightly compromised soft tissue, stable components, and a causative pathogen that is susceptible to an agent with activity against biofilm organisms [93].

At the time of reimplantation, different surgical techniques were described in the literature, including revision TAR and AA. 

Arthrodesis conversion represents one of the most common salvage procedures [94,95,96], in particular when facing a compromised general status of the patient, a vascular or soft-tissue impairment, uncontrolled diabetes, and bone stock loss [6,18,97,98]. It has been described either after a single or two-stage approach. Various surgical techniques and fixation methods are possible for TAR arthrodesis conversion: fusion can be achieved through screws, intramedullary nails [82,99,100,101], or Ilizarov frame [11,20,31,102,103]. In the case of severe bone loss after the prosthesis removal, bone allografts, autografts, or replacement materials (porous metals such as Trabecular Metal™) can be used to bridge the defect [104]. However, arthrodesis can be associated with leg length discrepancy, malunions, non-unions, and adjacent joint arthritis. In a systematic review, Gross et al. [96] analyzed the outcome of arthrodesis after TAR failure, showing an overall failure (non-union) rate of 10.6%. Moreover, Rahm et al. [81] claimed that the outcome after salvage AA seems to be less satisfactory (impaired life quality and reduced function, higher pain levels) compared to primary arthrodesis outcomes. 

Revision arthroplasty has been reported with success [105,106]. The use of dedicated revision components is generally suggested [97]. While Hintermann et al. [6] reported that medium-term results of revision arthroplasty after failed TAR were similar to those of after primary ankle replacement, in other studies patients have often complained of persistent chronic pain after revision surgery, requiring amputation in some cases. However, none of the studies focusing on revision TARs for PJIs assessed functional outcomes in detail.

Only a few studies described definitive cement spacer implantation as a treatment option for an infected TAR [30,86]. Despite some good results reported in the literature, this treatment has very limited indications and can rarely be considered as a definitive solution. Indications were usually represented by asymptomatic patients after a cement spacer insertion, medically unfit patients who do not desire any further revision surgery, or local tissue impairment. Ferrao et al. [30] reported a series of six infected TARs treated with retained antibiotic cement spacer as the definitive treatment. Good clinical outcomes were described after a mean follow-up of 20 months. Nevertheless, a conceptual error in PJI treatment could lie behind the choice of keeping a permanent spacer. In fact, antibiotic elution from cement spacers decreases over time, until the spacer remains an avascular foreign body that can be colonized by bacterial biofilms as for prosthetic implants [107]. 

In particularly difficult cases, transtibial amputation has been advocated [2,21,82,85]. Amputation is rarely a first-line treatment, but it should be considered in cases of persistent and active infection that are not controlled by medical or surgical treatment, vascular compromise, severe soft-tissue impairment, extensive bone loss, medical co-morbidities, or chronic pain [19].

An inherent limitation of our systematic review is the quality of the studies available. We were reliant on details provided by the included studies, which were largely retrospective case series and small case series. While specific analyses were not performed, we realize that there is inherent heterogeneity and bias between the individual included studies, which impacts our pooled averages. As a result, this limits the validity of our findings to some degree. The definition of revision was not clearly reported in all studies, and there was significant variability in the protocols used for revision surgery after PJI. 

In conclusion, while the prevalence of ankle PJI remains low, it is potentially one of the most devastating complications of TAR. Ankle PJI should be treated with aggressive debridement of an infection using careful soft-tissue-handling techniques. In any setting with chronic infection where salvage is reasonable, two-stage revision with culture-directed antibiotic spacer and antibiotic treatment for at least 6 weeks is recommended. However, this review highlights the lack of strong literature regarding TAR infections, thus highlighting the need for multicentric studies with homogeneous data regarding the treatment of ankle PJI to better understand outcomes. 

## Figures and Tables

**Table 1:** Characteristics of included studies. Epidemiology of TAR PJIs. PJI: Prosthetic Joint Infections; *n*: total number; NR: Not reported; NA: Not available.

Study	Patients (*n*)	PJI (*n*)	Incidence (%)	Age, Years (Mean)	Follow-Up, Months (Mean)	Type of Infection			
						Early	Late	Acute Hematogenous	NR
Myerson et al. [18]	613	19	3.10%	57	19	3	15	1	_
Kessler et al. [11]	511	34	6.65%	62	31	19	15	_	_
Patton et al. [19]	966	29	3.00%	55	55	14	7	_	8
Carlsson et al. [20]	100	4	4.00%	69	40	_	4	_	_
Kotnis et al. [21]	16	2	12.50%	56	12	_	2	_	_
Doets et al. [14]	93	5	5.38%	58	84	3	2	_	_
Wood et al. [22]	200	1	0.50%	60	46	_	1	_	_
Lee et al. [23]	50	1	2.00%	59	12	_	1	_	_
Saltzman et al. [24]	90	3	3.33%	63	6	_	3	_	_
Schutte et al. [25]	49	4	8.16%	57	28	_	_	4	_
V. D. Heide et al. [13]	58	6	10.34%	55	30	_	6	_	_
Young et al. [26]	1	1	NA	58	16	_	1	_	_
Henricson et al. [27]	93	4	4.30%	57	42	_	4	_	_
Reuver et al. [28]	64	3	4.69%	57	36	_	3	_	_
Devries et al. [29]	5	1	NA	67	17	_	1	_	_
Ferrao et al. [30]	6	6	NA	63	62	_	6	_	_
McCoy et al. [31]	7	3	NA	59	58	_	3	_	_
Rodrigues-Pinto et al. [32]	119	2	1.68%	56	35	_	2	_	_
Hintermann et al. [6]	117	9	7.69%	55	72	_	9	_	_
Oliver et al. [33]	245	4	1.63%	66	39	_	4	_	_
Richter et al. [34]	935	11	1.18%	62	110	_	_	_	11
Brunner et al. [35]	77	1	1.30%	47	85	_	1	_	_
Clough et al. [36]	200	1	0.50%	60	85	_	1	_	_
Adams et al. [37]	194	5	2.58%	64	4	2	3	_	_
Bennett et al. [38]	173	3	1.73%	60	24	1	2	_	_
Berlet et al. [39]	121	3	2.48%	62	12	_	3	_	_
Bianchi et al. [40]	62	1	1.61%	57	42	_	1	_	_
Borenstein et al. [41]	65	1	1.54%	64	16	1	_	_	_
Bouchard et al. [42]	87	1	1.15%	62	3.8	_	1	_	_
Lewis et al. [43]	249	5	2.01%	60	40	_	5	_	_
Loewy et al. [44]	138	4	2.90%	62	100	_	4	_	_
Mann et al. [45]	84	3	3.57%	61	108	3	_	_	_
Muir et al. [46]	178	3	1.69%	64	48	_	_	_	3
Mosca et al. [47]	73	1	1.37%	62	31	_	1	_	_
Buechel et al. [48]	50	2	4.00%	49	60	1	1	_	_
Claridge et al. [49]	28	4	14.29%	65	45	_	4	_	_
Lachmann et al. [50]	1600	14	0.88%	61	34	_	_	14	_
Wagener et al. [51]	13	1	7.69%	60	84	_	_	_	1
Usuelli et al. [52]	150	4	2.67%	53	12	_	3	1	_
Tan et al. [53]	20	1	5.00%	64	18	_	_	_	1
Strauss et al. [54]	11	2	NA	57	30	2	_	_	_
Cody et al. [55]	159	6	3.77%	63	20	_	_	_	6
Cody et al. [56]	538	5	0.93%	62	60	_	_	_	5
Daniels et al. [57]	229	3	1.31%	62	108	_	_	_	3
Nieuwe Weme et al. [58]	88	1	1.14%	57	60	_	1	_	_
Noelle et al. [59]	97	4	4.12%	63	36	_	_	_	4
Pangrazzi et al. [60]	104	3	2.88%	65	46	_	_	_	3
Pedersen et al. [61]	100	1	1.00%	59	65	_	1	_	_
Demetracopoulos et al. [62]	395	3	0.76%	62	40	_	3	_	_
Demetracopoulos et al. [63]	80	1	1.25%	67	40	_	1	_	_
Di Iorio et al. [64]	44	2	4.55%	56	120	_	_	_	2
Faber et al. [65]	51	1	1.96%	70	50	_	_	_	1
Giannini et al. [66]	76	1	1.32%	62	24	_	1	_	_
Gross et al. [67]	762	8	1.05%	63	13	_	8	_	_
Harston et al. [68]	149	1	0.67%	63	48	_	_	_	1
Heida et al. [69]	404	1	0.25%	65	NR	_	_	_	1
Henricson et al. [70]	324	14	4.32%	NR	NR	_	_	_	14
Hurowitz et al. [71]	62	3	4.84%	55	40	_	3	_	_
Jung et al. [72]	54	3	5.56%	63	30	_	3	_	_
Karantana et al. [73]	52	1	1.92%	62	80	_	1	_	_
Kerkhoff et al. [74]	67	2	2.99%	63	40	1	1	_	_
Koivu et al. [75]	34	3	8.82%	56	159	_	3	_	_
Koo et al. [76]	55	1	1.82%	70	60	_	1	_	_
Kraal et al. [77]	93	3	3.23%	58	120	1	1	_	1
Lagaay et al. [78]	94	1	1.06%	59	30	1	_	_	_
Halverson et al. [79]	5	1	NA	63	60	1	_	_	_
Kamrad et al. [80]	73	2	2.74%	55	NR	_	2	_	_
Rahm et al. [81]	23	6	26.09%	62	25	_	6	_	_
Berkowitz et al. [82]	24	2	8.33%	62	42	_	_	_	2
Bai et al. [83]	67	1	1.49%	56	38	_	1	_	_
Najefi et al. [84]	34	2	5.88%	58	58	_	_	2	_

**Table 2:** Characteristics of included studies. Treatment and outcomes. DAIR: Debridement Antibiotics and Implant retention; PJI: Prosthetic Joint Infection; NR: Not reorted.

Study	Patients	PJI	Timing of PJI, Months (Mean)	Treatment					Final Treatment					PJI Recurrence (%)
				DAIR	One Stage	Two Stage	Antibiotic	NR	Amputation	Prosthesis	Arthrodesis	Cement Spacer	NR	
Myerson et al. [18]	613	19	18	4	5	10	_	_	4	3	6	6	_	4 Recurrences (21%) 3 Reinfections (15.7%)
Kessler et al. [11]	511	34	NR	21	4	9	_	_	1	26	7	_	_	4 Recurrences (11.7%) 3 Reinfections (8.8%)
Patton et al. [19]	966	29	18	5	7	17	_	_	6	18	3	2	_	5 Recurrences (17.2%)
Carlsson et al. [20]	100	4	59	_	4	_	_	_	_	_	4	_	_	_
Kotnis et al. [21]	16	2	24	_	2	_	_	_	1	_	1	_	_	_
Doets et al. [14]	93	5	NR	4	_	1	_	_	_	4	1	_	_	_
Wood et al. [22]	200	1	NR	_	1	_	_	_	_	_	1	_	_	_
Lee et al. [23]	50	1	3	_	_	1	_	_	_	1	_	_	_	_
Saltzman et al. [24]	90	3	NR	_	2	1	_	_	_	_	2	1	_	1 Recurrence (33.3%)
Schutte et al. [25]	49	4	NR	_	3	1	_	_	_	1	3	_	_	_
V. D. Heide et al. [13]	58	6	25	3	3	_	_	_	1	3	2	_	_	_
Young et al. [26]	1	1	42	_	_	1	_	_	_	1	_	_	_	_
Henricson et al. [27]	93	4	NR	1	1	1	1	_	_	3	1	_	_	_
Reuver et al. [28]	64	3	NR	1	2	_	_	_	_	1	2	_	_	_
Devries et al. [29]	5	1	48	_	_	1	_	_	_	_	1	_	_	1 Recurrence (20%)
Ferrao et al. [30]	6	6	NR	_	_	6	_	_	_	_	_	6	_	_
McCoy et al. [31]	7	3	72	_	3	_	_	_	_	_	3	_	_	_
Rodrigues-Pinto et al. [32]	119	2	10	_	1	1	_	_	_	1	1	_	_	_
Hintermann et al. [6]	117	9	52	_	_	9	_	_	_	7	2	_	_	_
Oliver et al. [33]	245	4	20	_	4	_	_	_	_	_	4	_	_	_
Richter et al. [34]	935	11	NR	_	_	11	_	_	_	11	_	_	_	_
Brunner et al. [35]	77	1	96	_	1	_	_	_	_	_	1	_	_	_
Clough et al. [36]	200	1	80	_	1	_	_	_	_	_	1	_	_	_
Adams et al. [37]	194	5	NR	2	_	3	_	_	1	3	1	_	_	_
Bennett et al. [38]	173	3	NR	1	_	2	_	_	_	3	_	_	_	_
Berlet et al. [39]	121	3	NR	_	_	3	_	_	_	3	_	_	_	_
Bianchi et al. [40]	62	1	9	_	_	1	_	_	_	1	_	_	_	_
Borenstein et al. [41]	65	1	NR	1	_	_	_	_	_	1	_	_	_	_
Bouchard et al. [42]	87	1	NR	_	_	1	_	_	_	1	_	_	_	_
Lewis et al. [43]	249	5	NR	2	2	1	_	_	1	3	1	_	_	_
Loewy et al. [44]	138	4	17	_	2	2	_	_	_	2	2	_	_	_
Mann et al. [45]	84	3	13	3	_	_	_	_	_	3	_	_	_	_
Muir et al. [46]	178	3	NR	_	_	3	_	_	_	3	_	_	_	_
Mosca et al. [47]	73	1	3	_	_	1	_	_	_	1	_	_	_	_
Buechel et al. [48]	50	2	3	_	_	_	_	2	_	_	_	_	2	_
Claridge et al. [49]	28	4	NR	2	_	2	_	_	_	4	_	_	_	_
Lachmann et al. [50]	1600	14	43	14	_	_	_	_	1	9	4	_	_	1 Recurrence (7.14%)
Wagener et al. [51]	13	1	48	_	1	_	_	_	_	_	1	_	_	_
Usuelli et al. [52]	150	4	NR	2	_	_	2	_	_	4	_		_	_
Tan et al. [53]	20	1	NR	1	_	_	_	_	_	1	_	_	_	_
Strauss et al. [54]	11	2	1	_	2	_	_	_	_	_	2	_	_	_
Cody et al. [55]	159	6	13	_	_	_	_	6	_	_	_	_	6	_
Cody et al. [56]	538	5	NR	_	_	_	_	5	3	_	_	_	2	_
Daniels et al. [57]	229	3	NR	_	_	_		3	_	_	_	_	3	_
Nieuwe Weme et al. [58]	88	1	NR	_	_	_	_	1	_	_	_	_	1	_
Noelle et al. [59]	97	4	NR	_	1	3	_	_	_	3	1		_	_
Pangrazzi et al. [60]	104	3	NR	2	_	_	1	_	_	3	_		_	_
Pedersen et al. [61]	100	1	49	_	_	1	_	_	_	1	_	_	_	_
Demetracopoulos et al. [62]	395	3	NR	_	1	2	_	_	1	1	1	_	_	_
Demetracopoulos et al. [63]	80	1	NR	_	_	1	_	_	_	1	_	_	_	_
Di Iorio et al. [64]	44	2	NR	_	_	_	_	2	_	_	_	_	2	_
Faber et al. [65]	51	1	NR	_	_	_	_	1	_	_	_	_	1	_
Giannini et al. [66]	76	1	NR	_	_	1	_	_	_	1	_	_	_	_
Gross et al. [67]	762	8	NR	4	2	2	_	_	2	6	_	_	_	1 Recurrence (12.5%)
Harston et al. [68]	149	1	NR	_	_	_	_	1	_	_	_	_	1	_
Heida et al. [69]	404	1	NR	_	_	_	_	1	_	_	_	_	1	_
Henricson et al. [70]	324	14	NR	_	_	_	_	14	_	_	_	_	14	_
Hurowitz et al. [71]	62	3	NR	_	_	2	_	1	_	2	_	_	1	_
Jung et al. [72]	54	3	20	1	_	2	_	_	_	3	_	_	_	_
Karantana et al. [73]	52	1	NR	_	1	_	_	_	_	_	1	_	_	_
Kerkhoff et al. [74]	67	2	NR	1	_	1	_	_	_	2	_	_	_	_
Koivu et al. [75]	34	3	53	_	_	_	_	3	_	_	_	_	3	_
Koo et al. [76]	55	1	30	_	1	_	_	_	_	_	1	_	_	_
Kraal et al. [77]	93	3	NR	_	1	1	_	1	_	1	1	_	1	_
Lagaay et al. [78]	94	1	NR	_	_	_	1	_	_	1	_	_	_	_
Halverson et al. [79]	5	1	NR	1	_	_	_	_	_	1	_	_	_	1 Recurrence (100%)
Kamrad et al. [80]	73	2	NR	_	_	2	_	_	_	1	1	_	_	_
Rahm et al. [81]	23	6	33	_	6	_	_	_	_	_	6	_	_	_
Berkowitz et al. [82]	24	2	NR	_	2	_	_	_	_	_	2	_	_	_
Bai et al. [83]	67	1	6	_	1	_	_	_	_	1	_	_	_	_
Najefi et al. [84]	34	2	26	1	1	_	_	_	_	1	1	_	_	_

## Data Availability

The data presented in this study are available on request from the corresponding author.
